# Strain-Engineered Graphene Grown on Hexagonal Boron Nitride by Molecular Beam Epitaxy

**DOI:** 10.1038/srep22440

**Published:** 2016-03-01

**Authors:** Alex Summerfield, Andrew Davies, Tin S. Cheng, Vladimir V. Korolkov, YongJin Cho, Christopher J. Mellor, C. Thomas Foxon, Andrei N. Khlobystov, Kenji Watanabe, Takashi Taniguchi, Laurence Eaves, Sergei V. Novikov, Peter H. Beton

**Affiliations:** 1 School of Physics & Astronomy, University of Nottingham, Nottingham, NG7 2RD, UK; 2 School of Chemistry, University of Nottingham, Nottingham, NG7 2RD, UK; 3 The National Institute for Materials Science, Advanced Materials Laboratory, 1-1 Namiki, Tsukuba, Ibaraki 305-0044, Japan

## Abstract

Graphene grown by high temperature molecular beam epitaxy on hexagonal boron nitride (hBN) forms continuous domains with dimensions of order 20 μm, and exhibits moiré patterns with large periodicities, up to ~30 nm, indicating that the layers are highly strained. Topological defects in the moiré patterns are observed and attributed to the relaxation of graphene islands which nucleate at different sites and subsequently coalesce. In addition, cracks are formed leading to strain relaxation, highly anisotropic strain fields, and abrupt boundaries between regions with different moiré periods. These cracks can also be formed by modification of the layers with a local probe resulting in the contraction and physical displacement of graphene layers. The Raman spectra of regions with a large moiré period reveal split and shifted G and 2D peaks confirming the presence of strain. Our work demonstrates a new approach to the growth of epitaxial graphene and a means of generating and modifying strain in graphene.

The introduction of strain provides a route to modify both the electronic properties and phonon spectrum of graphene monolayers^1^,^2^,^3^,^4^,^5^. Local strains which form spontaneously when graphene is placed in an aligned configuration on a hexagonal boron nitride (hBN) surface can lead to the formation of an energy gap resulting in a high resistance state, a requirement for technological applications such as transistor action^6^,^7^,^8^,^9^,^10^,^11^,^12^,^13^. In addition, strain fields lead, for certain symmetries, to the formation of an energy gap which may be explained in terms of an equivalent pseudomagnetic field^3^,^14^. These observations have motivated attempts to introduce strain through various fabrication strategies such as the thermal shrinkage of attached films, and also the transfer of graphene to patterned surfaces^15^,^16^,^17^. Here we demonstrate that strained, epitaxial graphene may be grown directly on hBN using molecular beam epitaxy (MBE) leading to moiré patterns with large periodicity, topological defects and, locally, strongly anisotropic distortion. Our observations are consistent with the presence of strains of ~1% which also lead to a splitting and red-shift of the 2D peak of the Raman spectrum. Furthermore, we show that the strain distribution may be modified post-growth using the probe of an atomic force microscope; this leads to the formation of cracks which modify the moiré pattern and the Raman spectrum through a local contraction and, thus, physical displacement of the graphene layer.

Growth by MBE is realised through the direct sublimation of one or more source materials, in this case carbon, onto a substrate surface and allows independent control of growth temperature and deposition rate. MBE is widely used to grow conventional semiconductor device-grade materials, particularly those which incorporate heterojunctions, but has so far had little impact on the growth of graphene. For this relatively new material chemical vapour deposition (CVD) is much more widely used^18^, often in combination with the transfer of the grown material^19^,^20^ to a dielectric substrate. Over recent years a new generation of graphene-based devices incorporating heterojunctions between graphene and other two-dimensional materials have emerged. These devices include resonant tunnelling diodes^21^,^22^, photodetectors^23^,^24^ and light emitting devices^25^ and are typically constructed by mechanically stacking individual layers of graphene, hBN and layered semiconductors^26^. These developments have motivated studies of the direct growth of heterostructures incorporating high quality heterointerfaces between graphene and other materials, for example hBN, without the need for a sacrificial catalytic surface. There have been significant advances in the use of CVD towards this goal^12^,^27^,^28^,^29^,^30^ by adapting the method of Li *et al*.^18^ for growth of graphene on copper foils. However, attempts to grow graphene using MBE, which might be expected to be well-suited to the growth of high-quality heterojunctions, on hBN^31^,^32^, as well as several other substrates including metal foils^33^,^34^, SiC^35^ and sapphire^36^,^37^,^38^, have so far not provided a route to the growth of large area (micron scale) epitaxial material.

We have grown graphene by MBE on hBN flakes which are exfoliated from high-temperature high-pressure grown bulk hBN crystals^39^ and mounted on a sapphire substrate. Carbon is evaporated by heating a high-purity graphite filament and the substrate temperature during growth is extremely high for MBE, we estimate ~1500 °C, much higher than the value reported in previous MBE studies of graphene growth. Further experimental details are given in the Methods section; a growth time of ~4 hours is required to form a graphene monolayer on hBN. Figure 1a shows a large area image (see Methods) of a typical hBN flake acquired using atomic force microscopy (AFM) in which there are many bright features corresponding to three-dimensional aggregates with heights in the range 10–100 nm and widths 50–500 nm. Also present on the surface are several bright lines; these features are associated with the thermal cycling of hBN on sapphire and are present even with no carbon deposition (images of control samples of annealed hBN with no deposited carbon are included in the Supplementary Information, SI). The overall topography is similar to that reported by other groups and attributed to a combination of the growth of graphitic carbon and the presence of step edges, defects and wrinkles on the hBN surface^31^,^32^.

The regions between these aggregates, which have typical separation of ~1 μm, have a uniform contrast in Fig. 1a, but higher resolution AFM scans, for example Fig. 1b (an image of the marked area in Fig. 1a) reveal a regular hexagonal arrangement, that has not been observed previously in MBE-grown material, and which in this region is well ordered with a period of 13.2 ± 0.1 nm. These hexagonal networks are similar to moiré patterns which have been previously reported for aligned exfoliated graphene on hBN by Woods *et al*.^6^ who argue that, for small angles of misalignment between the principal axes of exfoliated graphene and an hBN substrate, a commensurate-incommensurate phase transition occurs, leading to a spatial variation of the lattice constant of the graphene. In the central areas of the hexagonal repeating unit, corresponding to low contrast regions in the AFM topographic image, it is argued that^6^ the graphene is stretched so that it is lattice matched to the hBN substrate. However, in the edge regions, which appear bright in our AFM images with a typical topographic height of 20 pm (see profile in Fig. 1i), the graphene is compressed so that N periods of graphene are overlaid on N-1 periods of hBN, with N reported^6^ to be ~10. Thus the bright edges correspond to the cores of dislocations which can be modelled as solitons within a description^6^ based on the Frenkel-Kontorova model.

For the exfoliated graphene studied by Woods *et al*.^6^, and also for graphene grown on hBN by CVD^12^,^29^ the period of the moiré pattern, λ_0_, is reported to be in the range 13.9–15 nm which is consistent with the value, 13.9 nm, expected for unstrained graphene (the moiré period λ_0_ = a^2^/δa, where a is the lattice constant of graphene, 0.245 nm, and δa is the difference between the lattice constants of hBN and graphene; we follow previous work^6^,^12^,^29^ and take δa/a = 1.8%).

The period of the hexagonal array in Fig. 1b, 13.2 nm, is close to that reported previously, but we also observe analogous patterns which have a much larger period and can be highly distorted. In Fig. 1c,d we show zoomed images of different parts of the flake shown in Fig. 1a where the periods are 22.0 and 26.4 nm respectively. The observation of a larger period, λ_s_, implies that the mismatch between the graphene and hBN has been reduced and, consequently, that the graphene is under tensile stress. The increase in the lattice constant averaged over a moiré unit cell is denoted Δa, and the strain, Δa/a, may be deduced from the increase in moiré period, Δλ = λ_s_–λ_0_ through the approximation Δa/a ≈ (δa/a) (Δλ/λ_s_). For the values observed in Fig. 1d this gives Δa/a = 0.9%.

In other areas of the flake (for example the zoomed areas in Fig. 1e–g) the hexagonal network undergoes significant distortion showing a local variation of the orientation of the lattice vectors of the moiré pattern and an asymmetry in the hexagonal unit cell. We also observe breaks in the graphene layer (Fig. 1h) across which there is an abrupt change in the moiré pattern from a regular hexagonal network with a period ~13 nm to a highly distorted pattern (these images also demonstrate that the distortion is not due to an imaging artefact). The height difference at these edges (Fig. 1j) is 0.3–0.4 nm, consistent with a single layer of graphene, and the width of the break is ~50 nm. Topological defects in the moiré pattern^40^ combined with a local distortion of the orientation and period of the hexagonal network are also observed; Fig. 1f shows the presence of a defect, an edge dislocation, where a row within the hexagonal pattern terminates. At these points the local co-ordination of the cells of the moiré pattern is 7 or 5 rather than 6 and the presence of defects leads to a long range curvature of the cellular network as shown in Fig. 1g. We also find large areas of up to ~2 μm which are largely free of both defects and aggregates (see SI).

We have considered two possible origins for the breaks in graphene such as those shown in Fig. 1h: firstly that they occur where two graphene domains have grown, but for some reason have not coalesced; or, alternatively, that a tear has been formed post-growth to release the stress in the graphene layer. In view of the highly parallel edges of the gap in Fig. 1h we propose that these are tears which are formed post-growth. As discussed below, we argue that the graphene layer is pinned by an array of nucleation sites (the three-dimensional aggregates) and the formation of cracks allows local relaxation of strain; in this case the graphene on one side of the crack relaxes to a value close to that expected for unstrained graphene, while on the other side the balance of stresses is modified leading to a strongly anisotropic strain distribution.

For a simple moiré pattern in which two rigid hexagonal networks are overlaid, we would not expect the distortion and topological defects observed in Fig. 1e–1h, but, following Woods *et al*.^6^, we attribute the bright edges of the hexagonal network to represent boundaries along which there is a local mismatch in the lattice constants of the hBN substrate and the graphene overlayer. Although it is natural, and may correspond to the lowest energy configuration, to assume that the boundaries run along the principal axes of the hBN and graphene, there is no reason *a prio*ri why the boundaries should not locally run in other directions. Note that a variation in orientation of the moiré network in CVD grown material has been reported previously^28^.

We have acquired AFM images in contact mode which show that the graphene lattice in regions on either side of the crack has the same orientation (within experimental error; see Fig. 1k) despite the difference in symmetry and period in the moiré patterns. Furthermore, contact mode images of the hBN surface exposed within the crack show that the graphene and hBN lattices are aligned. This confirms that the moiré pattern need not be aligned with the graphene lattice and that the growth is epitaxial. The highly anisotropic distortion observed in Fig. 1e thus implies that the graphene is anisotropically strained; in the most distorted region in Fig. 1h the cell within the hexagonal network has dimensions ~55 nm along two principal directions corresponding to a uniaxial strain of ~1.4%.

We have mapped the network of cracks across the complete 30 μm square area shown in Fig. 1a to reveal a domain structure as shown in Fig. 2a (the cracks are more clearly resolved in phase images). This confirms that there are crack-free regions which extend over lengths up to 20 μm (the largest unbroken domain marked as 2 in Fig. 2a), much larger than the typical separation of aggregates. To determine the variation of the period we have undertaken a systematic mapping of the graphene across the area highlighted in Fig. 2a (requiring the acquisition of 425 successive images over ~100 hours of continuous scanning). We extract, and map, the local period of the moiré pattern (see Fig. 2b,c).

The presence of strain is also expected to influence the Raman spectrum of the graphene and we have acquired a full two-dimensional Raman map of the flake in Fig. 1a (see Fig. 2d,e); in addition spectra with longer acquisition time, and lower noise, were acquired along the trajectory marked in Fig. 2e which cuts the boundary region between domains 1 and 2. Typical spectra are shown in Fig. 2e and confirm a systematic dependence of the 2D peak on moiré period; for λ_s_ = 26.3 nm the spectrum in the 2D region may be decomposed into three peaks. These consist of a high energy peak at 2682 cm^−1^ and two red-shifted peaks at 2583 cm^−1^ and 2527 cm^−1^. The peak at 2682 cm^−1^ is close to the value expected for monolayer and turbostratic graphene^41^,^42^, and is attributed to the presence of the three dimensional aggregates (see Fig. 1a) which are unavoidably present within the ~1 μm^2^ spot size of our Raman microscope (see Methods). This peak is present in all spectra with a near-constant energy but strong variation in amplitude, reflecting the inhomogeneous distribution of aggregates on the surface. The position of the red-shifted peaks shows a monotonic variation with λ_s_, strongly supporting the hypothesis that the graphene is strained; this has not been observed in previous reports of exfoliated or CVD graphene on hBN^12^,^29^,^43^.

As shown in Fig. 2d we also observe a dependence of the G peak on moiré period. The G and D’ peaks are observed at respectively 1581 cm^−1^ and 1615 cm^−1^ close to the expected values for graphene. However, we also observe a shoulder on the low energy side of the G peak which evolves into a more distinct feature which can be decomposed into two broad peaks centred on 1553 and 1527 cm^−1^ for λ_s_ = 26.3 nm. Other peaks present in the spectrum but not shown in Fig. 2d are a D peak at half the unshifted 2D peak, and a peak due to the hBN substrate. We have also confirmed that the group of 2D peaks are dispersive with excitation wavelength, while the set of G peaks is not dispersive, as expected^42^. We do not observe a partner D peak at half the wave number of the red-shifted 2D peak position, indicating that the strained graphene monolayer has low defect density, unlike the carbon aggregates. These additional Raman measurements are included in the SI. The amplitudes of the unshifted G and D’ peaks are correlated with that of the unshifted 2D peak at 2681 cm^−1^. These peaks are therefore associated, at least partially, with the presence of carbon aggregates.

To generate a Raman map we fit the 2D region of the measured spectrum at each point to the red, green and blue spectra shown in the inset to Fig. 2e (these model spectra are chosen to represent the peak corresponding to carbon aggregates (blue), the two red-shifted components for λ_s_ ~ 14 nm (green) and the red-shifted components for λ_s_ ~ 26 nm (red)); the false colour map in Fig. 1d is formed from the relative weights (determined by classical least squares (CLS) analysis) of the blue, green and red spectra which indicate the presence, respectively, of carbon aggregates, small moiré and large moiré periods. There is a clear correlation between the AFM and Raman map and we highlight the abrupt change in both moiré period and Raman spectra across the boundary between domains 1 and 2 (this corresponds to the crack shown in in Fig. 1h). Interestingly, the width of the relaxed domain with period ~13.2 nm is ~5 μm (see Fig. 2a,c). According to our model this has been formed by the physical displacement of the domain edge which was previously attached to the (now distorted) region of domain 2. Noting that the width of the crack is ~50 nm this would imply that the original strain in the layer was ~1%, consistent with our estimate from the moiré period in domain 2.

In Fig. 2f,g we plot the moiré period and the Raman peak positions along the trajectory marked by a line on the Raman and AFM maps. The Raman spectra were fitted using multiple Gaussian functions in order to deconvolve the peak positions of the overlapping bands. Also plotted is the anisotropy of the moiré pattern (expressed as g = (λ_max_/λ_min_−1) where λ_max/min_ are the maximum/minimum periods extracted locally; note that g = 0 for a non-distorted moiré pattern). These data, plotted in Fig. 2g confirm the spatial correlation of the Raman features and the moiré period. They also show that the distorted region (where g > 0) is localised to a region in domain 2 which is within ~2 μm of the crack forming the boundary with domain 1.

We also extract the dependence of the 2D peak positions on percentage strain, determined from the moiré period as discussed above (see Fig. 2g). Specifically, we find that the peaks shift at a rate of −119 ± 9 cm^−1^/% strain and −86 ± 6 cm^−1^/% strain. In addition, we observe a small shift even for Δλ = 0. It has previously been shown experimentally^1^ that the 2D peak shifts under uniaxial stress at a rate of −64 cm^−1^/%. The dependence of the red-shifted G peak is more difficult to determine from our data since the peaks are rather broad. Assuming a linear dependence on strain (see SI) we find a red-shift dependence of −37 cm ^−1^/% and −28 cm^−1^/% for the two additional components. However, from symmetry considerations we would not expect either the G or the 2D peaks of a freestanding graphene layer to be split for isotropic strain. We speculate that the split peak is due to interactions with the hBN substrate, for example a difference in local environment of carbon atoms in analogy with the formation of 2D_1_ and 2D_2_ features in graphite^42^; in fact it has recently been shown that the strain fields within a graphene/hBN heterojunction are complex with some regions of isotropic strain and some, close to the dislocation network, where the strain is expected to be uniaxial^13^. Overall, the Raman spectra presented here are consistent with the presence of strain, while revealing complexity of the vibrational spectra of these nanoscale heterostructures.

In Fig. 3 we show AFM images for varying coverages of graphene. At low fractional coverage of the hBN (Fig. 3a,b; growth time 1 hour) we see near-circular islands of graphene growing outward from the carbon aggregates which are present on the surface (we do not observe significant numbers of islands growing in the absence of an aggregate). On many of these islands a hexagonal moiré pattern is observed which in some cases is rather distorted. From such images we identify the aggregates as nucleation sites for graphene growth. We also observe the continuous merging, or coalescence, of two neighbouring islands (Fig. 3c); interestingly, for this particular example the moiré patterns on the two islands are not orientationally aligned and the pattern is discontinuous through the necking region which connects them.

As the carbon dosage is increased (Fig. 3d, growth time 4 hours; this is an intermediate coverage between Fig. 3a and Fig. 1) we see a near complete layer of graphene in which a highly distorted moiré pattern co-exists with small topographically bright dot-like features (typical width and height 10 and 0.2 nm respectively; indicating a different origin to the much larger aggregates discussed earlier). In the light of these results we propose that the graphene is nucleated at the carbon aggregates which form on the surface (Fig. 3a,b), then grows outward and coalesces with islands nucleated at neighbouring sites (Fig. 3c). Since the moiré patterns of islands nucleated at different sites can vary in their orientation and their registry with the substrate, there is inevitably a mismatch in the cellular patterns at the boundaries where they meet resulting in a disordered arrangement (Fig. 3d). We propose that further deposition of carbon results in a partial relaxation of the dislocation network towards a configuration which is more ordered, but in which some topological defects remain present.

We have confirmed that the cracks, such as in Fig. 1h, can be formed post-growth and can lead to strain relief by generating similar features using an AFM cantilever operated in contact mode. Figure 4 shows a region of the surface before and after the formation of a damaged track which is shown in Fig. 4c. We also observe narrow cracks with highly parallel edges and a width of 25 nm emanating from the damaged region (see Fig. 4c; zooms of a region before and after propagation of the crack are shown in Fig. 4d,e). These cracks were not present prior to the formation of the tear and, furthermore, after the formation of the crack the local moiré pattern was reduced from 22 nm to 17 nm and 14 nm on, respectively, the left and right of the tear (these values are averages of the periods in the three principal directions; note that the moiré pattern is anisotropically distorted both before and after propagation of the crack). The influence of the local tip damage thus propagates over large areas, modifying the strain distribution within the graphene; note that in this modification process one of the carbon aggregates has also been displaced and the crack runs continuously (highlighted by the red circle in Fig. 4a,b) under a neighbouring aggregate which implies that graphene is present under these 3D islands. Remarkably, the graphene edge has undergone a physical movement, through shrinkage, so that the process corresponds to a tip-induced mechanical actuation of the graphene.

We propose that the strain in the graphene layer is introduced during post-growth cooling and is maintained by a quasi-random distribution of nucleation sites which act to pin the material. The simplest explanation for the origin of the strain is a difference between the thermal coefficients of expansion of graphene, ∝_g_, and hBN, ∝_BN_. A tensile strain of ~1%, as observed here, implies that (∝_g_–∝_BN_), averaged over the relevant temperature range (300–1770 K) is positive with a value 8 × 10^−6^/K. The coefficient for hBN (the bulk value is relevant for our flakes) is −3 × 10^−6^/K at 300 K and remains negative, but with reduced magnitude^44^, up to 800 K. There is considerable divergence in the published values for ∝_g_; Bao *et al*.^45^ argue that ∝_g_ is negative at 300 K, but then increases to a positive value for a temperature, T > 350 K, while Yoon *et al*.^46^ find a negative value up to 400 K. Most recently, Linas *et al*. argued that ∝_g_ is positive over all measured temperatures in agreement with theoretical predictions^47^. For the maximum temperature (800 K) for which data are available^47^, (∝_g_–∝_BN_) ~3 × 10^−6^/K. This is lower than the average quoted above, but we emphasise that data are not available between 800 K and the growth temperature, 1770 K, and, given the uncertainty related to these quantities, thermally-induced strain is a plausible origin for the observed effects.

Graphene grown by CVD on hBN does not exhibit the large moiré pattern reported here and one possible reason for this difference is the pinning role of the nucleation sites in MBE growth; these aggregates are not observed in CVD growth. There are other differences, for example the growing edge of graphene is likely terminated by hydrogen in CVD growth^29^, while hydrogen should be absent during MBE growth leading to a more reactive edge and potentially stronger local interaction with the hBN substrate^32^. In addition, there is a potentially interesting interaction between the free edges of a growing island and the dislocation network. In Fig. 3b the dislocations intersect the free boundary at 90^o^; in fact we would not expect a bright dislocation edge to be formed parallel, and close to, a free boundary since the mismatch in lattice constants which occurs in these regions must be stabilised by compressive stress arising from the lattice-matched graphene on each side of the dislocation. This implies a complex evolution of the dislocation network due to finite size effects as the island continues to grow and a possible activated process for the introduction of new dislocation lines. Note that the introduction/elimination of a dislocation parallel to an island edge generates an advancement/retraction of the edge and therefore modifies the overall strain in the island. It is possible that thermally activated fluctuating displacements of the islands edge occur until neighbouring islands coalesce thus stabilising a strained configuration.

Our results demonstrate, for the first time, that MBE can be used to grow large areas of aligned graphene and also, unexpectedly, that the resulting material can be highly strained. Moreover, the strain, and physical properties of the graphene, can be modified through the generation of cracks using the probe of an AFM. This also leads to a physical displacement of the edges of graphene domains so the AFM actuates a nanoscale relative displacement of the grown layer, and, furthermore, gives rise to highly parallel edges which appear regular on a nanometre scale. The strongly anisotropic deformation close to a crack indicates the possibility of better controlled routes towards the engineering of more complex strain fields in graphene, for example through the lithographic formation of arrays of etched holes, or the formation of artificial pinning sites on hBN through nanofabrication prior to growth. Our work motivates further exploration of MBE growth as a route to the controlled introduction of strain into graphene layers for the generation of pseudomagnetic fields of order 10 T, novel electronic structure and new device paradigms.

## Methods

We use a custom-designed dual-chamber GENxplor MBE system (base pressure ~10^−10^  Torr) supplied by Veeco which is modified to reach growth temperatures of 1850 °C and is compatible with substrates up to 3 inches in diameter. In this system the substrates are mounted in a vertical configuration with the substrate heater mounted above the substrate. To deposit carbon we use a SUKO-63 sublimation source, essentially a high purity pyrolytic graphite filament which is Joule heated, supplied commercially by Dr. Eberl (MBE-Komponenten GmbH). As substrates we use exfoliated flakes of hBN from high-temperature high-pressure grown bulk hBN crystals^39^. The thicknesses of hBN flakes range from 10–100 nm and their lateral dimensions from 20–100 μm. These flakes are transferred onto 1 × 1 cm^2^ double sided polished sapphire (0001) substrates (SurfaceNet GmbH) and cleaned by immersion in toluene (99.9% CHROMASOLV for HPLC, Sigma Aldrich) overnight and heating at 400 °C for eight hours in a flow of 0.15 sl/min of Ar/H_2_ (95:5). The substrates are loaded on a Ta holder and following entry to the MBE system are pre-annealed at 400 °C for 0.5 hour prior to growth. Due to the use of the transparent sapphire substrate, pyrometer measurements give a reading only of the heater temperature. Instead we estimate the substrate temperature during growth using a thermocouple mounted in the substrate heater. The carbon deposition rate is estimated to be in the range 18–22 nm/hour from AFM measurements of the thickness of adsorbed carbon on the exposed sapphire regions of the substrate; note that the fraction of adsorbed carbon incorporated into epitaxial graphene on the adsorbed hBN flake is small, ~1%, so a growth time of ~4 hours is required to form a partial monolayer. The lifetime of the carbon source under these conditions is 2–6 hours so we are limited in our current work to monolayer growth. All of the samples discussed below were grown at the same substrate temperature which we estimate to be 1500 °C.

AFM images were acquired using an Asylum Research Cypher-S instrument operating in AC mode under ambient conditions and using Multi75AI-G cantilevers (Budget Sensors) supplied commercially by Windsor Scientific.

Raman spectra were recorded on a Horiba–Jobin–Yvon LabRAM Raman microscope, with a laser wavelength of 532 nm operating at low power (~4 mW) and a 600 lines/mm grating. The detector was a Synapse CCD detector. The Raman shift was calibrated using an Si(100) reference sample. The Raman CLS map was obtained by extracting the 2D region of each spectra in the map. The model spectra were selected from the dataset to best represent the different regions observed in the map. The presence of a small carbon aggregate peak (blue spectrum) in the red shifted spectra (green and red) was removed by subtracting the blue spectrum after applying a multiplier to it to ensure complete subtraction of the unwanted feature. CLS fitting to each spectrum in the map was performed using the Horiba Labspec 6 software.

## Additional Information

**How to cite this article**: Summerfield, A. *et al*. Strain-Engineered Graphene Grown on Hexagonal Boron Nitride by Molecular Beam Epitaxy. *Sci. Rep.* **6**, 22440; doi: 10.1038/srep22440 (2016).

## Supplementary Material

Supplementary Information

## Figures and Tables

**Figure 1:**
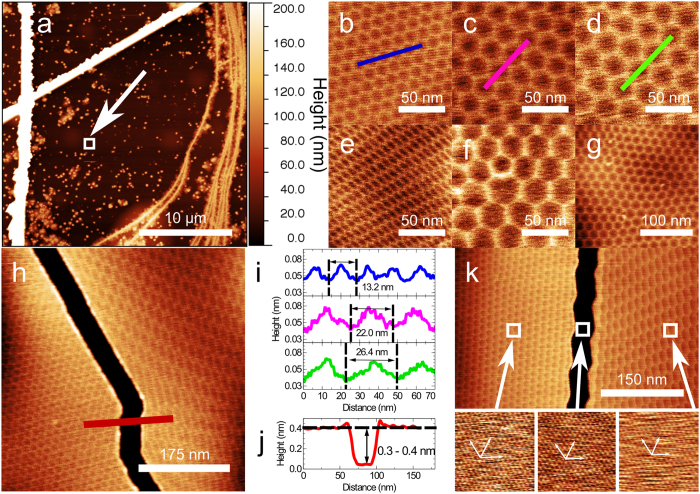
Topographic AFM images of graphene grown on hBN by MBE: (**a**) large area image showing large carbon deposits growing on defects on hBN; the straight lines running near vertically on the left of the image and diagonally across the image appear when thermally cycling the hBN and are not associated with carbon deposition while the meandering lines on the right of the image are due to growth at hBN terrace steps; (**b**) hexagonal moiré pattern with a period of 13.2 nm; (**c**) larger period (22.0 nm) moiré pattern; (**d**) 26.4 nm period moiré pattern; (**e**) distorted moiré pattern; (**f**) topological defect in moiré pattern; (**g**) long range distortion of moiré pattern due to topological defects; (**h**) crack in graphene bounded by moiré patterns with different period and anisotropic distortion; (**i,j**) profiles extracted from (**b–d and h,k**) (top) contact mode image of a crack in the graphene surface along with (bottom) graphene and hBN lattice images of the regions indicated by the arrows showing the orientation of the graphene and hBN lattices. The lattice images are 4.4 nm square.

**Figure 2:**
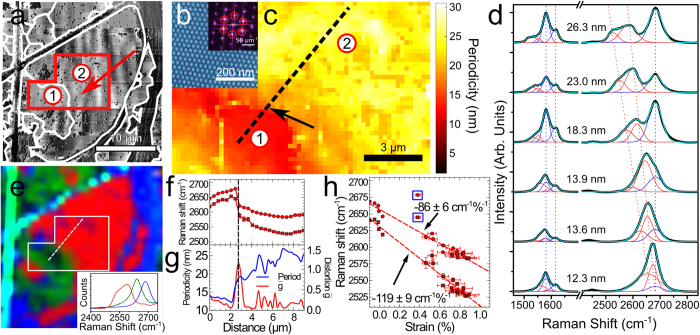
Correlation of Raman spectra and AFM images: (**a**) AFM phase image of the region shown in Fig. 1a with domain structure indicated by the white contours along with the locations of domains 1 and 2; (**b**) phase image of a position indicated by the arrows in (**a**) (inset) 2DFFT of b with first-order peaks corresponding to the moiré periodicity indicated by the red circles; (**c**) map of the periodicity across the region indicated by the red box in (**a**) showing the abrupt change in periodicity across the boundary between graphene domains 1 and 2; (**d**) selected Raman spectra showing the evolution of the red shifting G and 2D bands with increasing moiré periodicity indicated for each spectra; the black line is experimental data and the overlaid blue line is a Gaussian fit using, for the 2D peak, two components which are red-shifted–the red curves–and one–the purple curve–arising from the carbon aggregates; in the G region there are two new peaks together with the G and D’ peaks; (**e**) Raman CLS map of the hBN/graphene flake in a; blue areas indicate regions of high carbon aggregate concentrations, whilst the red and green approximate to the regions of higher and lower red shifted 2D bands, respectively; these are determined by fitting the spectrum at each point to the model spectra shown in the inset and , in more detail, in SI; (**f**) Raman 2D peak positions along the profile marked in (**c**,**e**); (**g**) profiles of the periodicity (blue line) and anisotropy (red line) across the same profile; (**h**) Raman shift of the 2D peaks as a function of strain for the points along the profile marked in (**c**); the highlighted points were acquired close to the boundary where the anisotropy is high and were not included in the calculation of the gradient.

**Figure 3:**
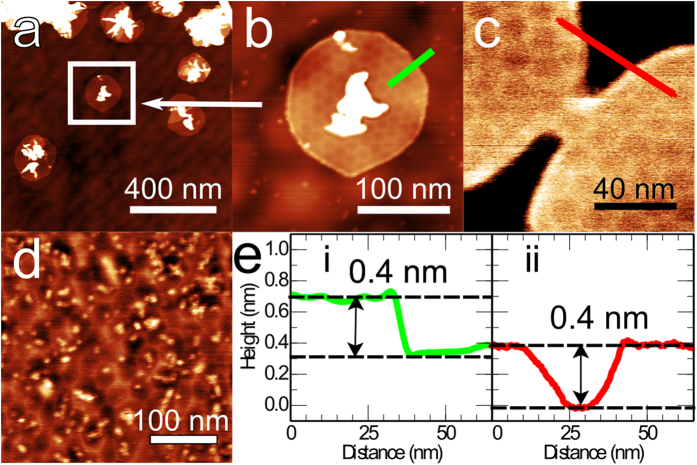
Intermediate stages of growth: (**a**) AC mode AFM images of graphene islands forming around carbon deposits on the hBN surface after 1 hour of growth; (**b**) island indicated by the box in (**a,c**) two graphene domains coalescing; (**d**) graphene on hBN after 4 hours of growth showing a distorted moiré pattern and deposits on hBN; (**e**) AFM height profiles across the regions indicated in (**b,c**).

**Figure 4:**
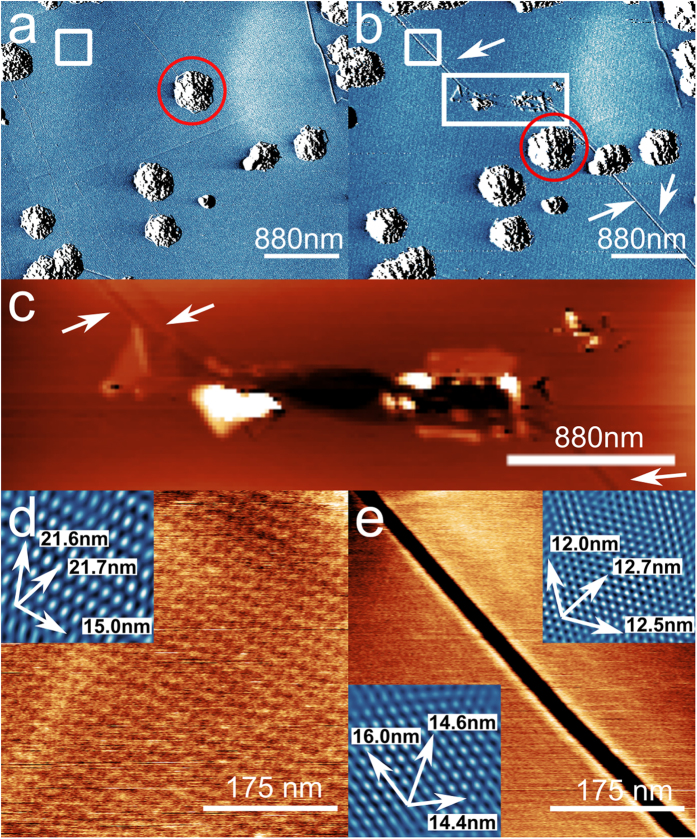
AFM-induced crack generation: (**a**) Amplitude channel AFM image of a region on the graphene surface before and (**b**) after damaging the sample by scanning the AFM tip in contact mode. The rectangular box in (**b**) shows the position of the damaged region and the arrows show the propagation of cracks away from the damaged area. In addition the red circle shows the displacement of a carbon deposit before and after the damage; (**c**) Detail of the damage introduced by the cantilever with arrows indicating cracks propagating away from the damage; (**d**) topographic image of the region indicated by the white box in (**a**); (inset) phase image showing the moiré period in different directions; (**e**) Height image of the same area after the formation of a crack as indicated by the box in (**b**); (insets) Filtered phase images of each side of the crack showing the reduction in moiré period in all directions indicating the strain has been relaxed.
