# Erratum: Strain-Engineered Graphene Grown on Hexagonal Boron Nitride by Molecular Beam Epitaxy

**DOI:** 10.1038/srep27047

**Published:** 2016-06-02

**Authors:** Alex Summerfield, Andrew Davies, Tin S. Cheng, Vladimir V. Korolkov, Yong Jin Cho, Christopher J. Mellor, C. Thomas Foxon, Andrei N. Khlobystov, Kenji Watanabe, Takashi Taniguchi, Laurence Eaves, Sergei V. Novikov, Peter H. Beton

Scientific Reports 6: Article number: 22440;10.1038/srep22440 Published online: 03012016; Updated: 06022016

The following statement has been omitted from the ‘Additional Information’ section of the HTML version of this Article:

“Data Availability: The images and spectra on which this paper is based may be publicly accessed and are stored at 10.17639/nott.35”.

The PDF version of this Article was correct at the time of publication.

