# Supplementary material for: Strain-Engineered Graphene Grown on Hexagonal Boron Nitride by Molecular Beam Epitaxy
Source: Sci Rep. 2016 Mar 1;6:22440. doi: 10.1038/srep22440 (PMC4772548; doi:10.1038/srep22440)
Supplement: Supplementary Information [file srep22440-s1.pdf]

Strain-Engineered Graphene Grown on Hexagonal Boron Nitride by Molecular Beam Epitaxy

Supplementary Information

Alex Summerfield[1], Andrew Davies[1,2], Tin S. Cheng[1], Vladimir V. Korolkov[1], YongJin Cho[1], Christopher J. Mellor[1], C. Thomas Foxon[1], Andrei N. Khlobystov[2], Kenji Watanabe[3], Takashi Taniguchi[3], Laurence Eaves[1], Sergei V. Novikov[1] and Peter H. Beton[1]

[1]School of Physics & Astronomy, University of Nottingham, Nottingham, NG7 2RD, UK

[2]School of Chemistry, University of Nottingham, Nottingham, NG7 2RD, UK

[3]The National Institute for Materials Science, Advanced Materials Laboratory, 1-1 Namiki, Tsukuba, Ibaraki 305-0044, Japan

1. AFM images of hBN flakes after annealing

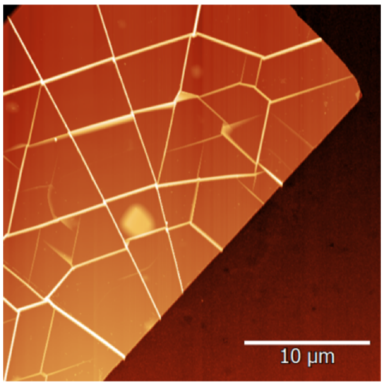

Figure S1. AFM image of a hBN flake on sapphire after heating to 1630˚C for 1 h showing the clear presence of wrinkles; no carbon has been deposited.

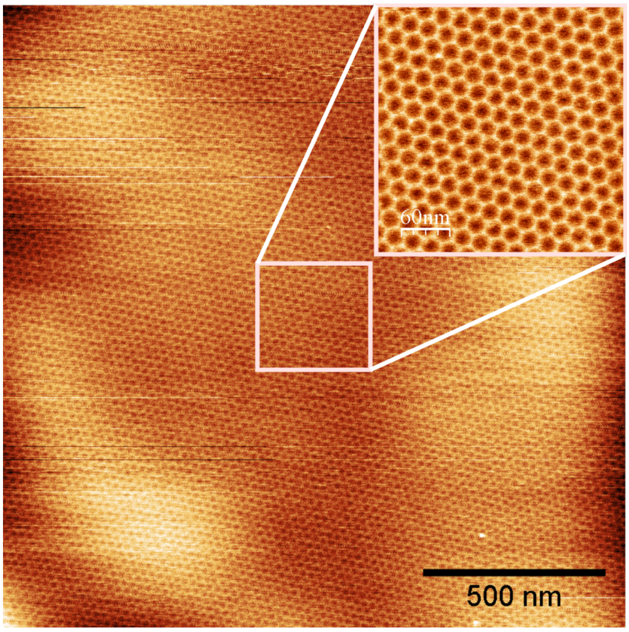

Figure S2. AFM image of a 1.6 µm square area of graphene free from bulky carbon deposits. (Inset) 300nm square Image of the centre of the main image clearly showing the moiré pattern on the graphene surface.

2. Optical micrograph of hBN flake

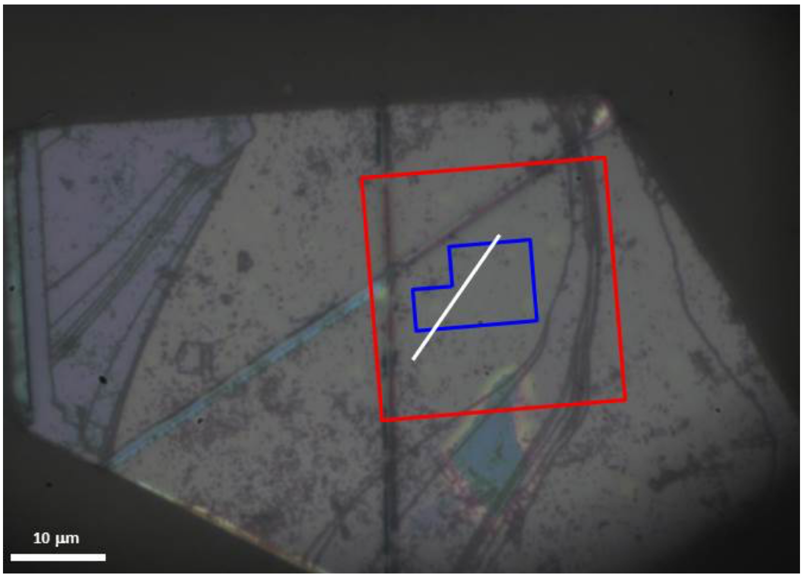

Figure S3. Optical image showing the regions where the Raman CLS map (red), Raman Line map (white), and AFM periodicity map (blue) were acquired.

3. Additional Raman data

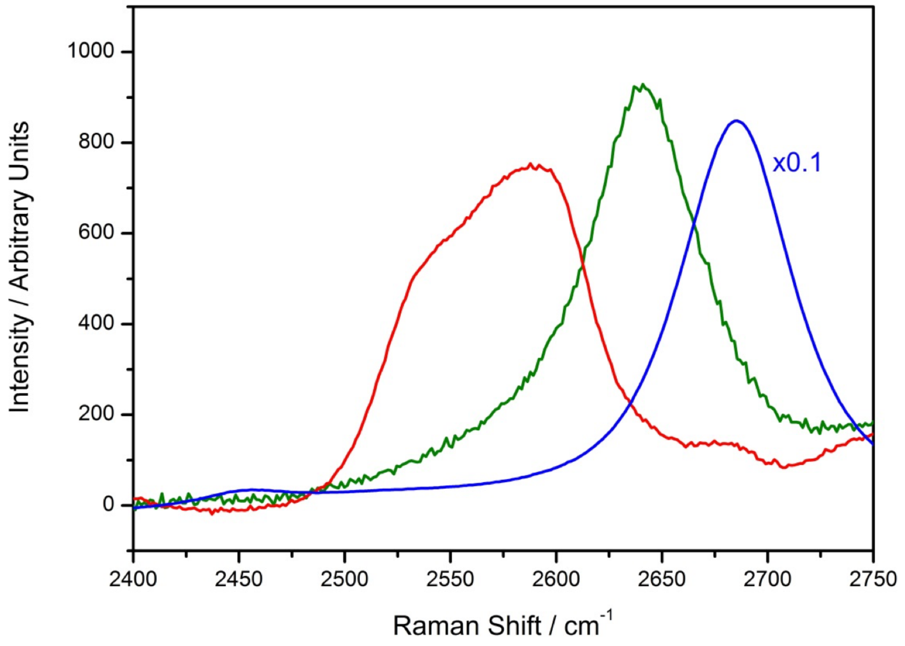

Figure S4. CLS Models for Raman Map

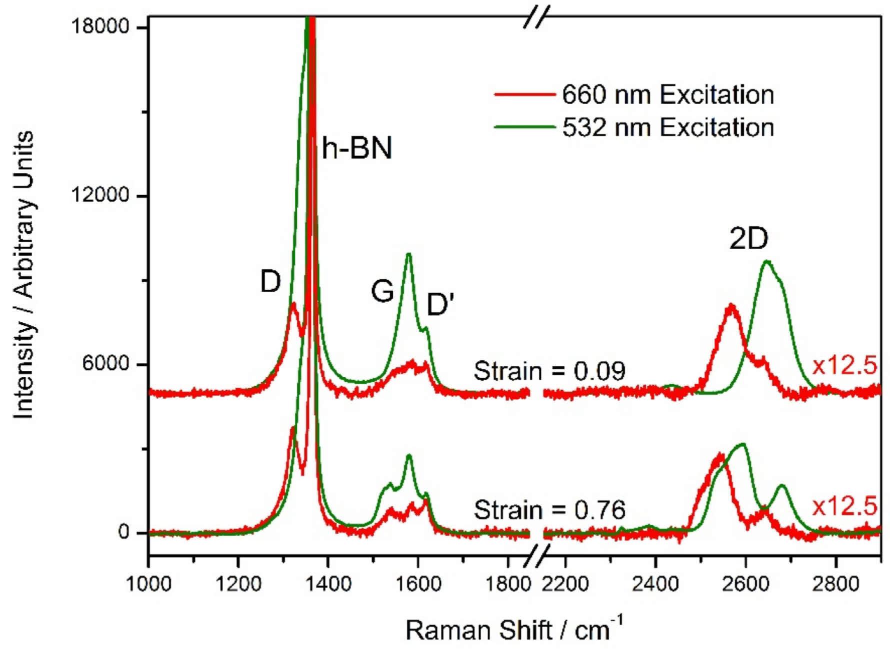

Figure S5. Comparison of Raman spectra acquired with different excitation wave lengths. The strain was inferred from the spectra recorded with an excitation wavelength of 532 nm by averaging the strain values obtained for the two red shifted 2D bands using the correlation graph in Fig. 2g.

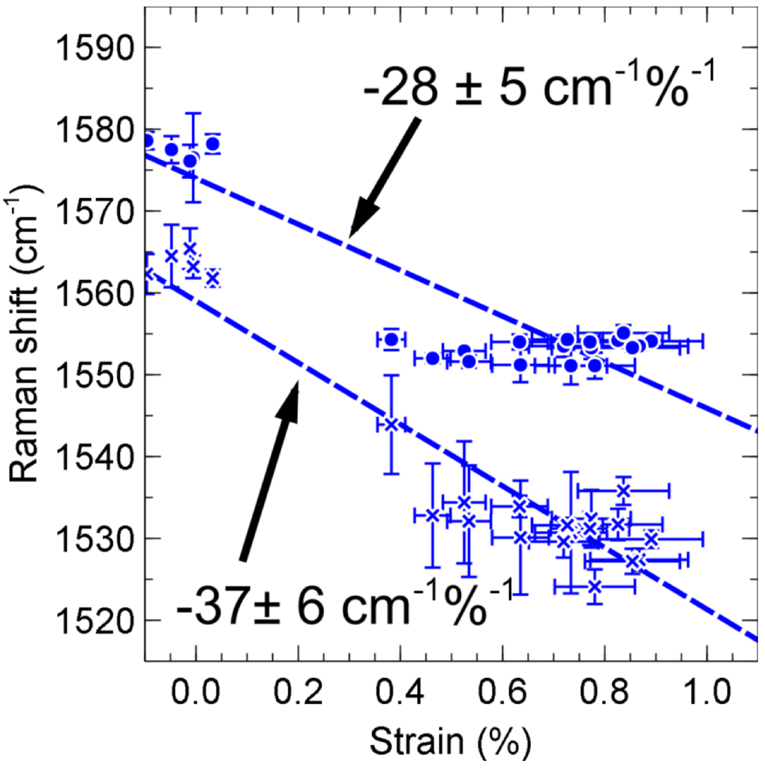

Figure S6. G peak positions as a function of strain.
